# Comparative pharmacokinetics, safety, and tolerability of two sources of ch14.18 in pediatric patients with high-risk neuroblastoma following myeloablative therapy

**DOI:** 10.1007/s00280-015-2955-9

**Published:** 2016-01-20

**Authors:** Araz Marachelian, Ami Desai, Frank Balis, Howard Katzenstein, Muna Qayed, Michael Armstrong, Kathleen A. Neville, Susan L. Cohn, Mark Bush, Rudy Gunawan, Allison Pecha Lim, Malcolm A. Smith, L. Mary Smith

**Affiliations:** Children’s Hospital Los Angeles, University of Southern California, 4650 Sunset Blvd, Los Angeles, CA 90027 USA; Children’s Hospital of Philadelphia, 3401 Civic Center Blvd, Philadelphia, PA 19104 USA; Vanderbilt University School of Medicine, 2200 Pierce Ave 397 PRB, Nashville, TN 37232-6310 USA; Aflac Cancer and Blood Disorders Center, Children’s Healthcare of Atlanta and Emory University School of Medicine, 1405 Clifton Rd NE, Atlanta, GA 30322 USA; Duke University, DUMC 102382, Durham, NC 27710 USA; Arkansas Children’s Hospital/University of Arkansas for Medical Sciences, 1 Children’s Way; Slot 512-23, Little Rock, AR 72202 USA; Department of Pediatrics, The University of Chicago, 900 E 57th Street, KCBD, Rm 5100, Chicago, IL 60637 USA; School of Pharmacy, Wingate University, 515 N. Main Street, Wingate, NC 28174 USA; Ionis Pharmaceuticals, 2855 Gazelle Court, Carlsbad, CA 92010 USA; United Therapeutics Corporation, 55 TW Alexander Drive, Research Triangle Park, NC 27709 USA; National Cancer Institute, 9609 Medical Center Dr, MSC 9739, Bethesda, MD 20892-9739 USA

**Keywords:** ch14.18, Dinutuximab, Pharmacokinetics, Safety, Tolerability, Unituxin

## Abstract

**Purpose:**

Dinutuximab (Unituxin™; ch14.18), a monoclonal antibody against disialoganglioside, improved survival as part of post-consolidation therapy for high-risk neuroblastoma. United Therapeutics Corporation (UTC) assumed ch14.18 production from the National Cancer Institute (NCI); this study evaluates pharmacokinetic comparability, safety, and tolerability of UTC and NCI products.

**Methods:**

In this randomized, two-sequence crossover study, 28 patients aged ≤8 years with high-risk neuroblastoma received equivalent ch14.18-UTC or ch14.18-NCI doses. Despite comparable protein content, nominal doses differed: 17.5 mg/m^2^/day (ch14.18-UTC) and 25 mg/m^2^/day (ch14.18-NCI). Patients received one product during therapy cycles 1 and 2, the other during cycles 3–5. Ch14.18 pharmacokinetic profile characterization used population modeling (NONMEM^®^ version 7.2). A two-compartment model with first-order distribution and elimination processes described pharmacokinetic data. Estimated product parameters were normalized to UTC nominal dose. For pharmacokinetic comparability, the final model was used to estimate exposure ratios (UTC/NCI) and associated 90 % confidence intervals (CIs) for area under the curve from time zero to infinity (AUC_inf_) and maximum concentration (*C*_max_). All comparisons were based on a standardized single-dose regimen (17.5 mg/m^2^ over 10 h).

**Results:**

Final-model pharmacokinetic parameters were similar to previously published ch14.18-NCI parameters and comparable for UTC and NCI products. Products’ systemic exposures were comparable, with 90 % CIs around ratios for AUC_inf_ (0.96; 90 % CI 0.88–1.04) and *C*_max_ (1.04; 90 % CI 0.98–1.11) within standard bioequivalence bounds (90 % CI 0.80–1.25). Products’ adverse events were similar and consistent with those previously reported.

**Conclusions:**

Equivalent actual ch14.18-UTC and ch14.18-NCI doses produced comparable exposures, with no notable safety or tolerability differences.

**Electronic supplementary material:**

The online version of this article (doi:10.1007/s00280-015-2955-9) contains supplementary material, which is available to authorized users.

## Introduction

Neuroblastoma, which is a tumor of the autonomic nervous system, accounts for approximately 7 % of cancers in children <15 years of age [1]; ~90 % of patients are <5 years of age at diagnosis [1]. The disease is heterogeneous, complex, and frequently aggressive [2]. At diagnosis, approximately 40 % of patients have high-risk disease [3], based on factors such as age, disease stage, and biologic markers (e.g., unfavorable histopathology, tumor amplification of *MYCN* oncogene) [4]. High-risk neuroblastoma is treated with dose-intensive chemotherapy and surgery, followed by myeloablative chemotherapy with autologous stem cell transplantation (ASCT), local radiation therapy, and maintenance with isotretinoin [5, 6]. Despite this intensive treatment, many patients relapse or have treatment-refractory disease, and 5-year event-free survival rates are ≤50 % [4, 7].

Disialoganglioside (G_D2_) is a surface glycolipid antigen that is strongly expressed on neuroblastoma tumor cells, with limited expression in normal human tissues [8]. G_D2_ is an important molecular target for immunotherapeutic approaches to treating neuroblastoma, and anti-G_D2_ monoclonal antibodies are efficacious in patients with high-risk neuroblastoma.

Dinutuximab (Unituxin™), formerly called ch14.18, is a murine–human chimeric anti-G_D2_ monoclonal antibody [9]. Initial trials demonstrated that ch14.18 at a dose of 25 mg/m^2^ infused over 10 h daily for 4 consecutive days could be incorporated into treatment regimens containing isotretinoin and the immunomodulators sargramostim and aldesleukin [10–12]. Subsequently, the Children’s Oncology Group (COG) conducted a randomized phase 3 clinical trial (ANBL0032) comparing ch14.18 administered with isotretinoin, sargramostim, and aldesleukin versus isotretinoin alone in patients with high-risk neuroblastoma who had responded to induction therapy, surgery, ASCT, and radiotherapy [12]. The trial demonstrated improved event-free survival (*p* = 0.01) and overall survival (*p* = 0.02) at 2 years on the immunotherapy arm [12]. Based on the results of this trial, dinutuximab received US Food and Drug Administration (FDA), and European Medicines Agency (EMA) approval for the treatment of high-risk neuroblastoma.

As part of a collaborative research agreement and development agreement (CRADA) with the National Cancer Institute (NCI), United Therapeutics Corporation (UTC) has licensed ch14.18 and assumed production. The nominal (i.e., labeled) dose of the UTC product (17.5 mg/m^2^) differs from the dose of the prior NCI product (25 mg/m^2^) because of a difference in the extinction coefficient used to determine the protein concentration during the manufacturing process. Despite the change in nominal dosing, the amount of antibody delivered per dose is equivalent for the two products. Corrections for this difference in the products’ nominal dose must be made when comparing dose-dependent pharmacokinetic parameters, such as clearance and volumes of distribution.

The primary objective of this study was to compare the pharmacokinetics of ch14.18 manufactured by these two independent facilities (i.e., NCI and UTC). The secondary objective was to compare the products’ safety and tolerability profiles.

## Materials and methods

### Study design

Study DIV-NB-201 was a phase 2 randomized, open-label, two-sequence crossover trial evaluating ch14.18 in patients with high-risk neuroblastoma scheduled to receive immunotherapy. The clinical trial was conducted in accordance with the ethical principles of the Declaration of Helsinki and the International Conference on Harmonization E6 Good Clinical Practice Guideline. The protocol was approved by the Institutional Review Board at each participating site, and the parents or guardians provided written informed consent with patient assent, as appropriate. Patients were randomized 1:1 to receive either ch14.18-UTC or ch14.18-NCI during cycles 1 and 2, followed by ch14.18 from the other product during cycles 3–5. Eligible patients were randomized between 56 and 105 days after ASCT. Randomization must have occurred after the completion of tumor assessments post-ASCT and radiotherapy, if applicable.

### Patients and treatment

Eligible patients were ≤8 years of age, had a diagnosis of high-risk neuroblastoma, and had completed standard induction therapy, surgery, myeloablative therapy and ASCT, and local radiotherapy to the primary tumor if indicated. Patients must have achieved a partial response or better per International Neuroblastoma Response Criteria (INRC) [13] at the primary site, soft tissue metastases, bone metastases, and bone marrow response at the pre-ASCT evaluation. Prior to enrollment, a determination of residual disease was performed, and patients could not have progressive disease per INRC except for protocol-specified bone marrow response to account for sampling errors. Patients were also required to have a Lansky performance status of ≥50 %; a total absolute phagocyte count ≥1000/μL; adequate renal, hepatic, cardiac, pulmonary, and central nervous system function; and a life expectancy of ≥2 months. Patients were excluded if they had received prior anti-G_D2_ antibody therapy or had prior vaccine therapy for the treatment of neuroblastoma. Patients were also excluded if they had received or planned to receive anticancer therapies, cytokines, or growth factors not included in the prescribed protocol therapy during the study or immunosuppressive drugs other than for acute allergic reactions and anaphylaxis during the study.

The study material manufactured by NCI used a theoretical extinction coefficient of 1.00 to calculate the concentration of antibody, whereas UTC material used an actual extinction coefficient of 1.41 to determine the antibody concentration. Thus, each 25 mg/m^2^ dose of ch14.18-NCI contains the same amount of ch14.18 as a 17.5-mg/m^2^ dose of ch14.18-UTC, making the respective dosing equivalent despite differences in the nominal doses. The dosing schema in this study is summarized in Table 1. During the first five cycles, patients received ch14.18-UTC or ch14.18-NCI intravenously (IV) over 10–20 h daily for four consecutive days repeated every 28 days, with one product administered during cycles 1 and 2 and the other product during cycles 3, 4, and 5. All patients received sargramostim IV or subcutaneously (SC) (250 mcg/m^2^/day for 14 days) on cycles 1, 3, and 5 prior to, during, and following ch14.18, and aldesleukin IV (3 MIU/m^2^/day for 4 days as a continuous infusion for the first week, followed by 4.5 MIU/m^2^/day for 4 days as a continuous infusion concurrently with ch14.18 for the second week) during cycles 2 and 4. In addition, all patients received six cycles of isotretinoin over 14 days (80 mg/m^2^/day orally twice daily for >12 kg and 2.67 mg/kg orally twice daily for ≤12 kg) after completion of ch14.18 therapy.

**Table 1:** Dosing schema and pharmacokinetic assessment schedule

Cycles 1, 3, and 5 (24 days in duration)																
Cycle day	0	1	2	3	4	5	6	7	8	9	10	11	12	13	14	15–24
Sargramostim		**X**	**X**	**X**	**X**	**X**	**X**	**X**	**X**	**X**	**X**	**X**	**X**	**X**	**X**	
ch14.18					**X**	**X**	**X**	**X**								
Isotretinoin												**X**	**X**	**X**	**X**	**X**
Pharmacokinetic assessments (cycles 1 and 3 only)	**X** ^**a**^			**X** ^**b,c**^	**X** ^**c**^	**X** ^**c**^	**X** ^**c**^	**X** ^**d**^		**X** ^**d**^	**X** ^**d**^	**X** ^**d**^			**X** ^**e**^	**X** ^**e**^

Cycles 2, 4, and 6 (32 days in duration)																
Cycle day (cycles 2 and 4)	0	1	2	3	4	5	6	7	8	9	10	11	12–14	15–28	29–32	
Aldesleukin		**X**	**X**	**X**	**X**				**X**	**X**	**X**	**X**				
ch14.18									**X**	**X**	**X**	**X**				
Isotretinoin														**X**		
Pharmacokinetic assessments (cycles 2, 4, and 6)		**X** ^**f**^							**X** ^**g**^			**X** ^**h**^			**X** ^**i**^	

^a^Day 0, up to 3 days prior to initial dose of sargramostim. ^b^ End of infusion, within 15 min of completion (days 3 and 59), preinfusion (day 59). ^c^ End of infusion, within 15 min of completion (days 4–6, 60–62). ^d^ 10–14 h post-completion of ch14.18 infusion (days 7 and 63). ^e^ Single sample between days 9–11, 14–17, 65–67, and 70–73. ^f^ Single sample, prior to aldesleukin (days 24 and 80). ^g^ Preinfusion of the first ch14.18 dose (days 31 and 87). ^h^ Immediately following the fourth daily ch14.18 infusion (day 90). ^i^ End of study within 2 weeks of the final isotretinoin dose (cycle 6, day 163)

In addition to study treatment, supportive care measures, including the use of narcotics and other concomitant medications, were required for the treatment of anticipated toxicities.

### Assessments

Pharmacokinetic blood samples for the determination of ch14.18 plasma concentrations were obtained at 22 time points over the course of the treatment (Table 1). Samples were drawn prior to the first dose of sargramostim on cycle 1 and the first ch14.18 infusion on cycle 3; after the end of each daily ch14.18 infusion; and 10–14 h, 3–5 days, and 8–11 days after the fourth ch14.18 infusion on cycles 1 and 3. Sampling during cycles 2 and 4 occurred prior to aldesleukin, prior to the first ch14.18 dose, and at the end of the fourth daily ch14.18 infusion on cycle 4 only. A final sample was obtained at study end, within 2 weeks of the final isotretinoin dose on cycle 6. Blood samples for the analysis of human anti-chimeric antibody (HACA) were obtained prior to ch14.18 dosing in each cycle. Plasma from each sample was isolated by centrifugation, frozen, and shipped to Burleson Research Technologies, Morrisville, NC, for storage. Samples were shipped to BioAgilytix, Durham, NC, where ch14.18 and HACA plasma concentrations were measured using validated assays: a sandwich immunoassay employing an electrochemiluminescence platform to measure ch14.18, and a Meso Scale Discovery electrochemiluminescent assay (Meso Scale Diagnostics, Rockville, MD) with a lower limit of quantification of a titer of 10 to measure HACA.

Because of the study complexity and data limitations, a model-based approach, rather than a traditional noncompartmental bioequivalence analysis, was used to assess the pharmacokinetic comparability of ch14.18-UTC and ch14.18-NCI. Detailed pharmacokinetic data from nine patients with high-risk neuroblastoma enrolled in a previous pharmacokinetic study of ch14.18-NCI at the Children’s Hospital of Philadelphia by Desai [14] were used to develop a structural population pharmacokinetic model. The final model was a two-compartment model with first-order distribution and elimination processes. To account for the effects of body size on pharmacokinetic parameters, actual body weight was included as a predetermined allometric covariate on all clearance and volume of distribution parameters. Estimated pharmacokinetic parameters included clearance from the central compartment (CL), distributional clearance (*Q*), volume of the central compartment (V1), volume of the peripheral compartment (V2), steady-state volume of distribution (*V*_ss_), first-order elimination rate constant (Kel), and first-order distribution rate constants (central-to-peripheral [Kcp]; peripheral-to-central [Kpc]). The final structural model for the data from the prior Desai study was then used to estimate pharmacokinetic parameters for ch14.18-UTC and ch14.18-NCI in the formal comparability study (DIV-NB-201). This staged analysis approach was taken to avoid the potential for inflated alpha error associated with iterative model development using the formal comparability data from DIV-NB-201.

Because calculations of some pharmacokinetic parameters are dependent on the nominal dose of drug, differences in the nominal doses of ch14.18-UTC and ch14.18-NCI will affect pharmacokinetic parameter estimates. In the Desai study [14], pharmacokinetic parameter estimates were based on nominal dosing units of ch14.18-NCI. Therefore, when comparing dose-dependent pharmacokinetic parameters generated using UTC and NCI nominal doses, appropriate corrections were made.

To formally assess the pharmacokinetic bioequivalence of the UTC and NCI products, maximum concentration (*C*_max_) and area under the curve from time zero to infinity (AUC_inf_) were calculated using the population pharmacokinetic parameter estimates of CL and V from DIV-NB-201 and appropriate closed-form equations for *C*_max_ and AUC_inf_. Variability in exposure estimates was captured by sampling the posterior distribution of pharmacokinetic parameter estimates using the NONMEM^®^ Markov Chain Monte Carlo methodology. To allow for valid comparisons, all calculations were based on a standardized single dose of 17.5 mg/m^2^ infused over 10 h, using nominal UTC doses. Pharmacokinetic bioequivalence was assessed by calculation of ratios (UTC/NCI) for AUC_inf_ and *C*_max_ with 90 % confidence interval (CI) of the ratios. AUC_inf_ was the primary comparability end point. Bioequivalence was established if the 90 % CIs for the exposure ratios were completely contained within accepted bioequivalence bounds (0.80–1.25).

Safety assessments included adverse event reporting, physical examinations, clinical laboratory assessments, and treatment-related changes in electrocardiograms (ECGs). Safety analyses were performed on all patients receiving at least one study drug dose. No inferential statistical analyses of safety data were planned.

## Results

Twenty-eight patients were enrolled, and 14 patients were randomized to each treatment sequence. Patient characteristics are summarized in Table 2 with no differences in demographics by sequence. A summary of concomitant medications used by patients during cycles 1–5 is presented in Supplemental Table 1. Patients’ completion or discontinuation of study therapy is summarized in Table 3. One patient was excluded from the pharmacokinetic analysis because of a neutralizing antibody response that interfered with measurement of ch14.18 concentration. All 28 patients received at least one dose of ch14.18 and were included in the safety analysis. Seven patients discontinued the study prior to completion of the planned six cycles; reasons were disease progression (*n* = 2), adverse events (*n* = 2), or withdrawal of consent (*n* = 1). Two patients received cycles 1–5 in the USA and returned to their home country to complete cycle 6. Ch14.18 was administered over a median duration of 11 h (range 10–20 h).

**Table 2:** Patient demographic and baseline characteristics

Characteristic	Sequence 1 (*n* = 14) (UTC/NCI)	Sequence 2 (*n* = 14) (NCI/UTC)
Mean age at randomization (range) (years)	4 (2–7)	4 (1–9)
Male gender, *n* (%)	8 (57)	8 (57)
Ethnicity, *n* (%)		
Hispanic	4 (29)	2 (14)
Not Hispanic	10 (71)	12 (86)
Race, *n* (%)		
White	12 (86)	11 (79)
Asian	0	1 (7)
Black/African American	2 (14)	1 (7)
Unknown	0	1 (7)
Pre-ASCT response, *n* (%)		
Complete response	5 (36)	3 (21)
Very good partial response	5 (36)	4 (29)
Partial response	4 (29)	7 (50)
Number of ASCT, *n* (%)		
Single	13 (93)	14 (100)
Tandem^a^	1 (7)	0
Prior chemotherapy, *n* (%)	14 (100)	14 (100)
Radiotherapy, *n* (%)	12 (86)^b^	13 (93)
Cancer-related surgery, *n* (%)	12 (86)^c^	11 (79)

*ASCT* autologous stem cell transplantation, *NCI* National Cancer Institute, *UTC* United Therapeutics Corporation

^a^Patients were required to undergo ASCT (first transplant for tandem transplant patients) within 9 months after starting the first induction chemotherapy for high-risk neuroblastoma. In addition, patients were required to enroll in the study within 105 days post-ASCT (date of second transplant for tandem patients) such that study day 0 (first dose of sargramostim) occurred within 110 days post-transplantation

^b^Radiotherapy may have been waived for patients who either had a small adrenal mass that was completely resected initially or who never had an identifiable primary tumor

^c^Patients may not have had an identifiable primary tumor

**Table 3:** Patients’ completion or discontinuation of study therapy

Disposition	Sequence 1 (UTC/NCI), *n* (%)	Sequence 2 (NCI/UTC), *n* (%)
Safety population, *n*	14	14
Pharmacokinetic population, *n*	13^a^	14
Completed all study therapy	9 (64)	12 (86)
Discontinued study therapy		
Cycle 1	1 (7)	0
Cycle 2	0	1 (7)
Cycle 3	2 (14)	0
Cycle 4	0	1 (7)
Cycle 5	2 (14)^b^	0
Reason for study discontinuation		
Disease progression	1 (7)	1 (7)
Adverse event	1 (7)^c^	1 (7)^d^
Consent withdrawn	1 (7)	0
Moved out of country	2 (14)^b^	0

*NCI* National Cancer Institute, *UTC* United Therapeutics Corporation

^a^One patient excluded because of interfering human anti-chimeric antibodies, for the pharmacokinetic assay

^b^Patients completed cycles 1–5 and went on to complete scheduled course of isotretinoin in their country

^c^Patient discontinued during cycle 3 due to serum sickness

^d^Patient discontinued during cycle 2 due to neuropathy

### Immunogenicity

Six of 27 patients had detectable HACA during the study. Only one patient (17 %) had a pharmacokinetic-neutralizing response (detected in cycle 3) and was therefore excluded from the pharmacokinetic analysis.

### Pharmacokinetics

**Fig. 1:**
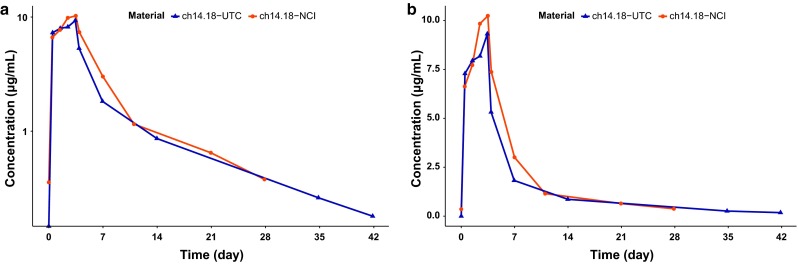
Representative semilog (**a**) and linear (**b**) concentration–time profiles from a single patient for ch14.18-UTC and ch14.18-NCI

Representative concentration time profiles for ch14.18-UTC and ch14.18-NCI from a single patient are presented in Fig. 1a (semilog), b (linear). A comparison of the pharmacokinetic profiles indicates similar exposures for both products. Population mean concentration time profiles are shown in Supplemental Figure 1. Summary statistics of post hoc pharmacokinetic parameters are presented in Table 4 for each product, separately and combined. Dose-dependent pharmacokinetic parameters were normalized to the nominal ch14.18-UTC dose. Clearance, volumes of distribution, and rate constants were equivalent for the NCI- and UTC-manufactured products.

**Table 4:** Summary of individual post hoc pharmacokinetic parameter estimates for ch14.18

Parameter, mean (SD)	ch14.18-UTC (*n* = 26)	ch14.18-NCI (*n* = 25)	ch14.18-UTC and ch14.18-NCI combined (*n* = 27)
CL (L/d)	0.683 (0.307)	0.75 (0.32)	0.709 (0.315)
CL (L/d/m^2^)	1.09 (0.457)	1.17 (0.455)	1.12 (0.457)
*Q* (L/d)	0.767 (0.163)	0.956 (0.213)	0.857 (0.198)
*Q* (L/d/m^2^)	1.20 (0.026)	1.46 (0.023)	1.32 (0.056)
V1 (L)	1.43 (0.403)	1.36 (0.42)	1.40 (0.404)
V1 (L/m^2^)	2.23 (0.29)	2.05 (0.28)	2.15 (0.28)
V2 (L)	3.94 (1.1)	3.76 (1.09)	3.85 (1.07)
V2 (L/m^2^)	6.10 (0.42)	5.65 (0.40)	5.87 (0.39)
*V*_ss_ (L)	5.38 (1.47)	5.12 (1.49)	5.25 (1.45)
*V*_ss_ (L/m^2^)	8.32 (0.57)	7.70 (0.57)	8.03 (0.54)
Kel (1/d)	0.489 (0.211)	0.569 (0.232)	0.520 (0.221)
Kcp (1/d)	0.552 (0.073)	0.723 (0.103)	0.630 (0.090)
Kpc (1/d)	0.199 (0.018)	0.259 (0.021)	0.228 (0.018)

*CL* clearance from the central compartment, *Kcp* first-order distribution rate constant (central-to-peripheral), *Kel* first-order elimination rate constant, *Kpc* first-order distribution rate constant (peripheral-to-central), *NCI* National Cancer Institute, *Q* distributional clearance, *UTC* United Therapeutics Corporation, *V1* volume of the central compartment, *V2* volume of the peripheral compartment, *V*_ss_ steady-state volume of distribution

Following standardized single-dose regimens, population pharmacokinetic estimates for AUC_inf_ were 431 µg·h/mL for ch14.18-UTC and 413 µg·h/mL for ch14.18-NCI (ratio = 1.04; 90 % CI 0.98–1.11). Population pharmacokinetic estimates for *C*_max_ were 6.57 µg/mL and 6.88 µg/mL, respectively (ratio = 0.96; 90 % CI 0.88–1.04). The 90 % CIs for exposure ratios of AUC_inf_ and *C*_max_ were contained within the standard bioequivalence bounds (0.80–1.25), consistent with comparable exposure between products.

### Safety

All 28 randomized patients were included in the safety analyses and had at least one treatment-related adverse event (TRAE) overall and at least one TRAE attributed to ch14.18. Overall, a total of 1945 TRAEs were reported, with most being grades 1–3. The most commonly reported TRAEs included pyrexia (100 %), hypoalbuminemia (96 %), hypokalemia (96 %), hyponatremia (82 %), cough (75 %), increased alanine aminotransferase (ALT) (68 %), anemia (68 %), hypocalcemia (68 %), pain (68 %), pruritus (68 %), increased aspartate aminotransferase (AST) (64 %), hypertriglyceridemia (64 %), and abdominal pain (61 %). Although differences between products were seen for individual adverse events, evaluation over the entire study showed no notable differences in TRAE incidence by manufacturer, either overall or attributable to ch14.18. Pain-related TRAEs were generally similar between treatment sequences; the most commonly reported pain-related events in the ch14.18-UTC and ch14.18-NCI groups were pain (59 and 44 %), abdominal pain (48 and 41 %), and pain in extremity (33 and 41 %), respectively. Pain-related TRAEs were most commonly reported during cycles 1, 2, and 4 (93, 74, and 71 %, respectively). Allergic-type adverse events were also generally similar between ch14.18-UTC and ch14.18-NCI groups, with the most commonly reported events (i.e., ≥15 % of patients) including urticaria (30 vs. 26 %), peripheral edema (15 vs. 11 %), pruritus (63 vs. 52 %), and rash (26 vs. 26 %), respectively, and occurred most frequently in cycles 1, 2, and 4.

Table 5 summarizes grade 3 or higher TRAEs considered by the investigator to be attributable to ch14.18 in ≥10 % of patients. The most common grade ≥3 events were pyrexia, anemia, hypokalemia, and hyponatremia, with no discernible differences between study drugs. Other safety assessments, including clinical laboratories, physical examinations, and ECGs, were generally consistent between UTC- and NCI-manufactured ch14.18.

**Table 5:** Grade 3 or higher treatment-related adverse events (≥10 %)

Adverse events, *n* (%)	ch14.18-UTC (*n* = 27)	ch14.18-NCI (*n* = 27)
≥1 Adverse event	22 (81.5)	23 (85.2)
Pyrexia	13 (48.1)	12 (44.4)
Anemia	6 (22.2)	9 (33.3)
Hypokalemia	7 (25.9)	7 (25.9)
Hyponatremia	5 (18.5)	5 (18.5)
Platelet count decreased	4 (14.8)	5 (18.5)
ALT increased^a^	4 (14.8)	1 (3.7)
Lymphocyte count decreased	2 (7.4)	3 (11.1)
Pain-related adverse events		
Pain	5 (18.5)	2 (7.4)
Pain in extremity	2 (7.4)	3 (11.1)
Abdominal pain	2 (7.4)	3 (11.1)
Hypocalcemia	3 (11.1)	2 (7.4)
Hypotension	2 (7.4)	3 (11.1)
Neutrophil count decreased	2 (7.4)	3 (11.1)
Hypoxia	1 (3.7)	3 (11.1)
Urine output decreased	3 (11.1)	1 (3.7)

*ALT* alanine aminotransferase, *NCI* National Cancer Institute, *UTC* United Therapeutics Corporation

^a^ALT increases were transient

## Discussion

The NCI-manufactured ch14.18 was used in the pivotal randomized phase 3 trial that demonstrated the efficacy of ch14.18 combined with sargramostim, aldesleukin, and isotretinoin administered as continuation therapy for high-risk neuroblastoma [12]. This randomized crossover study comparing equivalent doses of ch14.18-UTC (17.5 mg/m^2^) and ch14.18-NCI (25 mg/m^2^) was conducted to confirm equivalence of the pharmacokinetic and safety profiles of the two products.

Pharmacokinetic parameters were estimated by fitting a two-compartment pharmacokinetic model to individual concentration–time data. The products were formally compared using the results of a model-based bioequivalence analysis, which showed equivalent systemic exposures as measured by the AUC_inf_ for ch14.18-UTC and ch14.18-NCI, and 90 % CIs about the geometric least-squares mean ratios for AUC_inf_ and *C*_max_ within standard bioequivalence bounds (90 % CI 0.80–1.25). Clearance, volume of distribution, and rate constants were also equivalent between the UTC and NCI products.

For this analysis, dose-dependent pharmacokinetic parameters were derived using the ch14.18-UTC nominal dose of 17.5 mg/m^2^. Historical parameters, such as CL and volumes of distribution, for the ch14.18-NCI material were derived using a 25-mg/m^2^ dose and require a correction factor of 0.7 for comparison with ch14.18-UTC material.

Data from an independent study (CHP1002) were used to develop a pharmacokinetic model for ch14.18. This model was then used to estimate pharmacokinetic parameters for the comparability study (DIV-NB-201). This approach was taken to avoid the potential for inflated alpha error associated with iterative model development based on the formal comparability data (DIV-NB-201). A model-based approach (rather than a traditional noncompartmental bioequivalence analysis) was chosen to assess the pharmacokinetic comparability of ch14.18-UTC and ch14.18-NCI because of data limitations and the complexity of DIV-NB-201. The pharmacokinetic parameters derived from the population model for the CHP1002 study were comparable to those previously published by Desai et al. [14]. The Desai analysis was also based on a two-compartment structural model; however, the methodology used for parameter estimation differed from the current analysis, which utilized a population pharmacokinetic approach. In addition, Desai et al. used different pharmacokinetic software (MLAB; Civilized Software, Silver Spring, MD). Despite these differences, mean pharmacokinetic parameters for the CHP1002 study were comparable between the Desai analysis and the population model. When pharmacokinetic parameters are expressed in terms of nominal ch14.18-NCI dosing units, estimates for the Desai analysis and the population pharmacokinetic analysis, respectively, are similar (CL, 2.1 vs. 1.9 L/d/m^2^; Vl, 2.2 vs. 1.9 L; Kel, 0.026 vs. 0.027 1/h; Kcp, 0.021 vs. 0.023 1/h; and Kpc, 0.010 vs. 0.015 1/h).

Overall, the TRAEs (including allergic-type events) observed in this study were consistent with those reported in other studies in which ch14.18 was administered with growth factors and cytokines [12, 15]. The most commonly reported adverse events in >60 % of patients, regardless of manufacturer, were pyrexia, hypoalbuminemia, hypokalemia, hyponatremia, cough, increased ALT, anemia, hypocalcemia, pain, pruritus, increased AST, hypertriglyceridemia, and abdominal pain.

The ANBL0032 study demonstrated that approximately 17 % of patients treated with ch14.18 develop HACA, with <5 % having a neutralizing antibody response in a biological assay (data on file, United Therapeutics Corporation). In this study, 6/27 (22 %) of patients had confirmed HACA, and only one patient was excluded from the pharmacokinetic analysis due to a neutralizing antibody response.

In summary, the current analysis confirms that the ch14.18-UTC dose of 17.5 mg/m^2^ is comparable to the ch14.18-NCI dose of 25 mg/m^2^ in terms of systemic exposure and with no notable safety and tolerability differences.

## Electronic supplementary material

Supplementary material 1 (DOCX 148 kb)
