# Supplementary material for: Comparative pharmacokinetics, safety, and tolerability of two sources of ch14.18 in pediatric patients with high-risk neuroblastoma following myeloablative therapy
Source: Cancer Chemother Pharmacol. 2016 Jan 20;77:405–12. doi: 10.1007/s00280-015-2955-9 (PMC4747995; doi:10.1007/s00280-015-2955-9)
Supplement: Supplementary file 1 — Supplementary material 1 (DOCX 148 kb) [file 280_2015_2955_MOESM1_ESM.docx]

**Supplemental Table 1** Summary of most common^a^ concomitant medications by class used during cycles 1-5

| Medication class/generic name^b, c^ | Cycle, n (%) | | | | |
| --- | --- | --- | --- | --- | --- |
|  | 1  N = 28 | 2  N = 27 | 3  N = 25 | 4  N = 24 | 5  N = 23 |
| **Analgesics^d^**  Morphine  Nalbuphine  Paracetamol | 28 (100)  23 (82)  11 (39)  28 (100) | 25 (93)  18 (67)  6 (22)  20 (74) | 23 (92)  17 (68)  3 (12)  18 (72) | 21 (88)  16 (67)  4 (17)  18 (75) | 19 (83)  15 (65)  5 (22)  15 (65) |
| **Anesthetics**  Lidocaine | 10 (36)  10 (36) | 4 (15)  3 (11) | 8 (32)  8 (32) | 2 (8)  1 (4) | 4 (17)  4 (17) |
| **Antibacterials for systemic use**  Cefepime  Ceftriaxone | 24 (86)  6 (21)  12 (43) | 26 (96)  10 (37)  13 (48) | 14 (56)  4 (16)  8 (32) | 18 (75)  4 (17)  14 (58) | 10 (44)  3 (13)  2 (9) |
| **Antiemetics and antinauseants**  Ondansetron | 15 (54)  15 (54) | 13 (48)  13 (48) | 8 (32)  8 (32) | 8 (33)  8 (33) | 5 (22)  5 (22) |
| **Antihistamines for systemic use^e^**  Diphenhydramine  Hydroxyzine | 26 (93)  23 (82)  12 (43) | 21 (78)  19 (70)  8 (30) | 19 (76)  14 (56)  8 (32) | 19 (79)  17 (71)  7 (29) | 18 (78)  13 (57)  6 (26) |
| **Anti-inflammatory and antirheumatic products**  Ibuprofen | 6 (21)  6 (21) | 13 (48)  13 (48) | 7 (28)  7 (28) | 7 (29)  7 (29) | 2 (9)  2 (9) |
| **Blood substitutes and perfusion solutions**  Albumin  Potassium  Red blood cells | 18 (64)  8 (29)  5 (18)  7 (25) | 17 (63)  12 (44) 7 (26)  6 (22) | 14 (56)  8 (32) 2 (8)  4 (16) | 13 (54)  8 (33)  6 (25)  4 (17) | 10 (44)  6 (26)  2 (9)  3 (13) |
| **Diuretics**  Furosemide | 11 (39)  11 (39) | 18 (67)  18 (67) | 7 (28)  7 (28) | 10 (42)  9 (38) | 6 (26)  6 (26) |
| **Drugs for acid-related disorders**  Ranitidine | 9 (32)  5 (18) | 9 (33)  7 (26) | 11 (44)  8 (32) | 7 (29)  6 (25) | 5 (22)  4 (17) |
| **All other therapeutic products**  Naloxone | 4 (14)  4 (14) | 5 (19)  5 (19) | 6 (24)  6 (24) | 4 (17)  4 (17) | 4 (17)  4 (17) |

^a^Reported by ≥25% of all patients across any cycle during cycles 1-5.

^b^Medications summarized by World Health Organization Drug Dictionary preferred name.

^c^Medications were summarized during the cycle in which they were initiated (ie, if a patient started a medication in cycle 1 and remained on this medication through cycle 5, it would have been summarized only in cycle 1. Conversely, if the patient had started a medication in cycle 1, discontinued its use in cycle 1, and then restarted the medication in cycle 2, it would have been summarized in both cycles 1 and 2).

^d^Patients were required to receive concomitant analgesics with ch14.18 administration.

^e^Patients were required to receive concomitant antihistamines with ch14.18 administration.

**Supplemental Fig. 1** Population mean concentration-time profiles for ch14.18-UTC and ch14.18-NCI. The population mean concentration-time profiles for ch14.18-UTC and ch14.18-NCI were

generated based on final PK parameter estimates from NONMEM^®^ and using a standardized

dosing regimen of 17.5 mg/m^2^ infused over 10 hours. NCI = National Cancer Institute; PK = pharmacokinetic; UTC = United Therapeutics Corporation.
